# A Rare Case of Telecanthus-Hypospadias Syndrome in a Pediatric Patient

**DOI:** 10.7759/cureus.37411

**Published:** 2023-04-10

**Authors:** Jayant Vagha, Ajinkya Wazurkar, Keta Vagha, Sham Lohiya, Ashish Varma

**Affiliations:** 1 Department of Pediatrics, Jawaharlal Nehru Medical College, Datta Meghe Institute of Higher Education and Research, Wardha, IND

**Keywords:** oral surgery, hypertelorism, multisystem, opitz syndrome, hypospadias, telecanthus

## Abstract

Hypertelorism and hypospadias are the main characteristics of telecanthus-hypospadias syndrome; however, it can also include other midline structural anomalies, such as cleft lip and palate, cryptorchidism, congenital heart problem, laryngotracheal cleft, esophageal fistula, and irregular scrotum. Here, we describe an eight-year-old male who was brought to us for cleft lip repair, but upon evaluation, the other listed anomalies were discovered. He had hypertelorism, hypospadias, a ventricular septal defect, and a history of cryptorchidism. A multidisciplinary approach involved pediatricians, oral surgeons, cardiologists, and pediatric surgeons. The patient underwent surgery for first-stage hypospadias correction and was advised to follow up for additional surgery and maintenance procedures before being discharged. We wish to report this case with the aim to enlighten budding pediatricians and surgeons about this rare syndrome.

## Introduction

Telecanthus-hypospadias syndrome is associated with widely spaced inner ocular canthi and hypospadias, which can be of varying degrees [1]. This syndrome is also known as Opitz syndrome. The syndrome includes several abnormalities, including genital abnormalities such as cryptorchidism, hypospadias, and bifid scrotum, as well as facial abnormalities including hypertelorism, prominent forehead, broad nasal bridge, and anteverted nares [2]. More than 50% of individuals had cleft lip or cleft palate involvement, compared to less than 1% who have midline brain defects (such as Dandy-Walker malformation and agenesis or hypoplasia of the corpus callosum and/or cerebellar vermis), congenital heart problems, or imperforate anus [2]. Males are affected and take a more dangerous course than females, who often take a more benign course and are mostly recognized because of the affected male family member [3]. Here, we present a unique case of an eight-year-old male who had a cleft lip and hypospadias and was later found to have telecanthus-hypospadias syndrome on clinical examination.

## Case presentation

**Figure 1:**
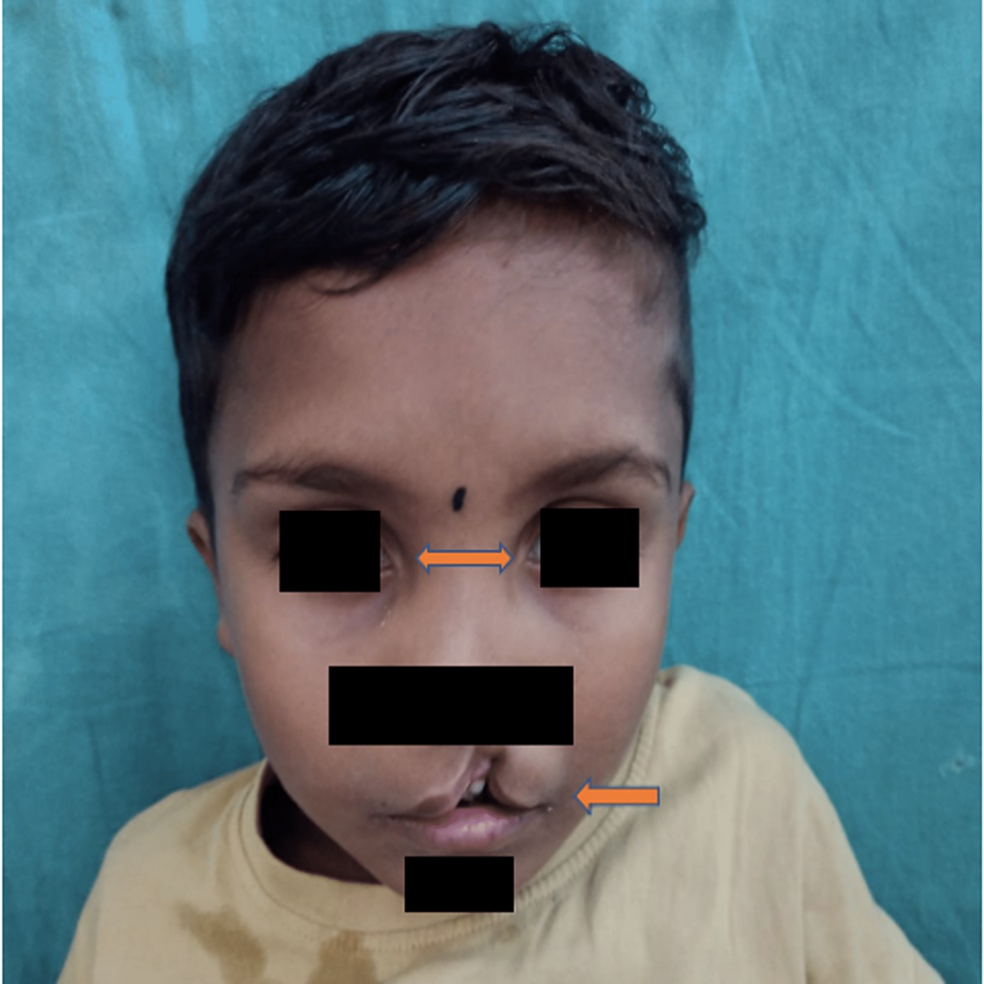
Clinical image of the patient showing widely spaced inner canthus suggestive of telecanthus, depressed nasal bridge, and a cleft lip Captured by JV

An eight-year male was brought to our tertiary care hospital in central India with complaints of cleft lip and hypospadias. He was operated on for left-sided undescended testis and left-sided inguinal hernia four years back for which orchidopexy and herniotomy were done. There was no developmental delay. On examination, his pulse rate was 84 beats/minute, respiratory rate was 20 breaths/minute, and blood pressure was 100/60 mmHg. The patient had a normal throat with no deformity noted on examination. A neurological examination did not reveal any deficit. He had significant facial features such as a prominent forehead, depressed nasal bridge, hypertelorism, anteverted nares, and cleft lip (Figure 1).

**Figure 2:**
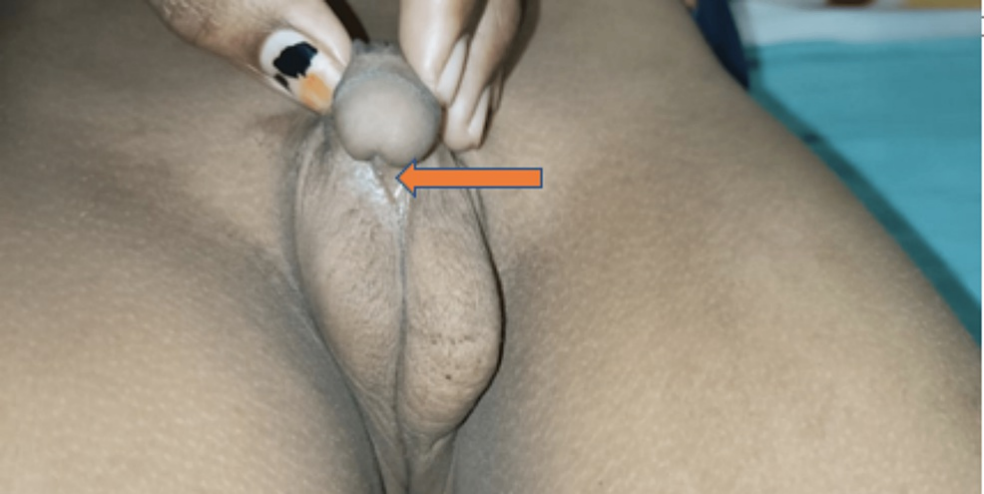
Clinical image of the patient showing proximal hypospadias with significant chordee Captured by JV

The chest of the patient was normal with no structural abnormality. He also had proximal hypospadias with significant chordee (Figure 2).

**Figure 3:**
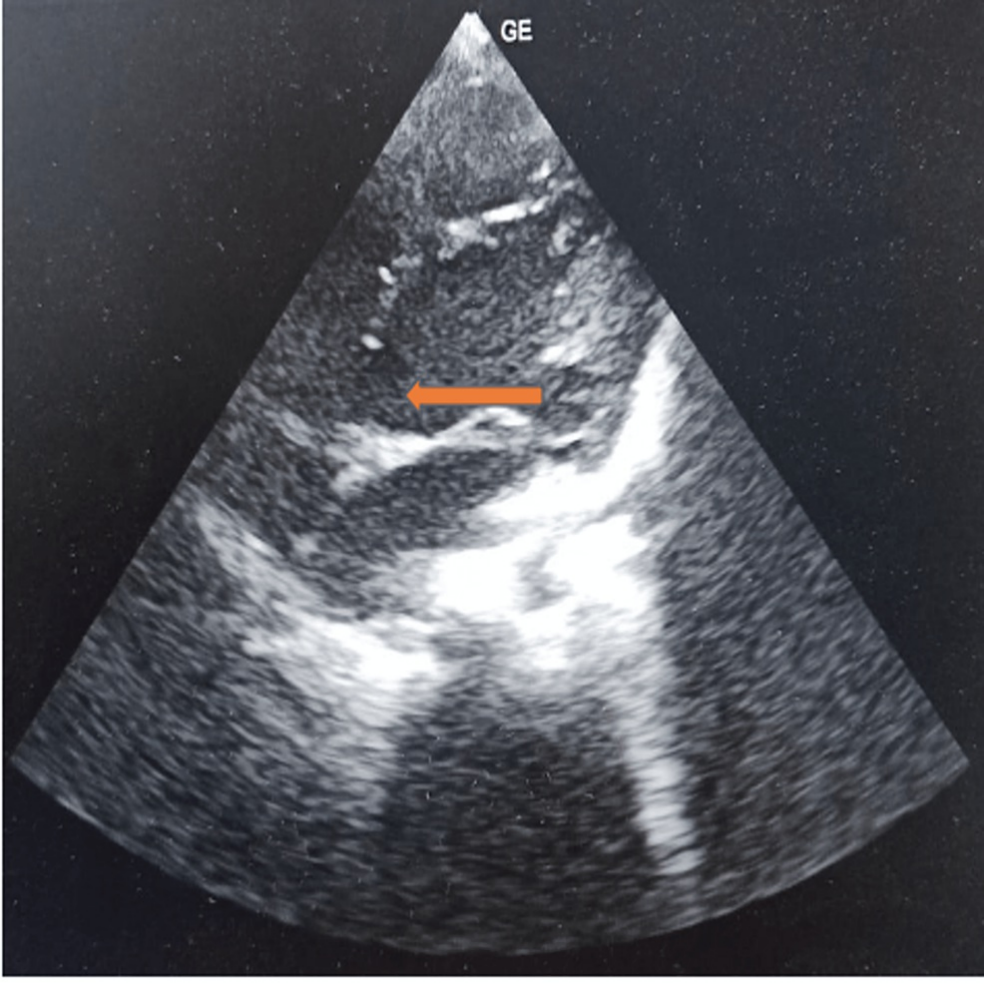
Echocardiography of the patient showing a 6 mm inlet type of ventricular septal defect on an apical four-chamber view Captured by JV

On auscultation, a pansystolic murmur was heard at the parasternal region in the fourth and fifth left intercostal space mitral with hyperdynamic apex impulse. His echocardiography showed a 6 mm inlet type of ventricular septal defect on an apical four-chamber view (Figure 3).

**Figure 4:**
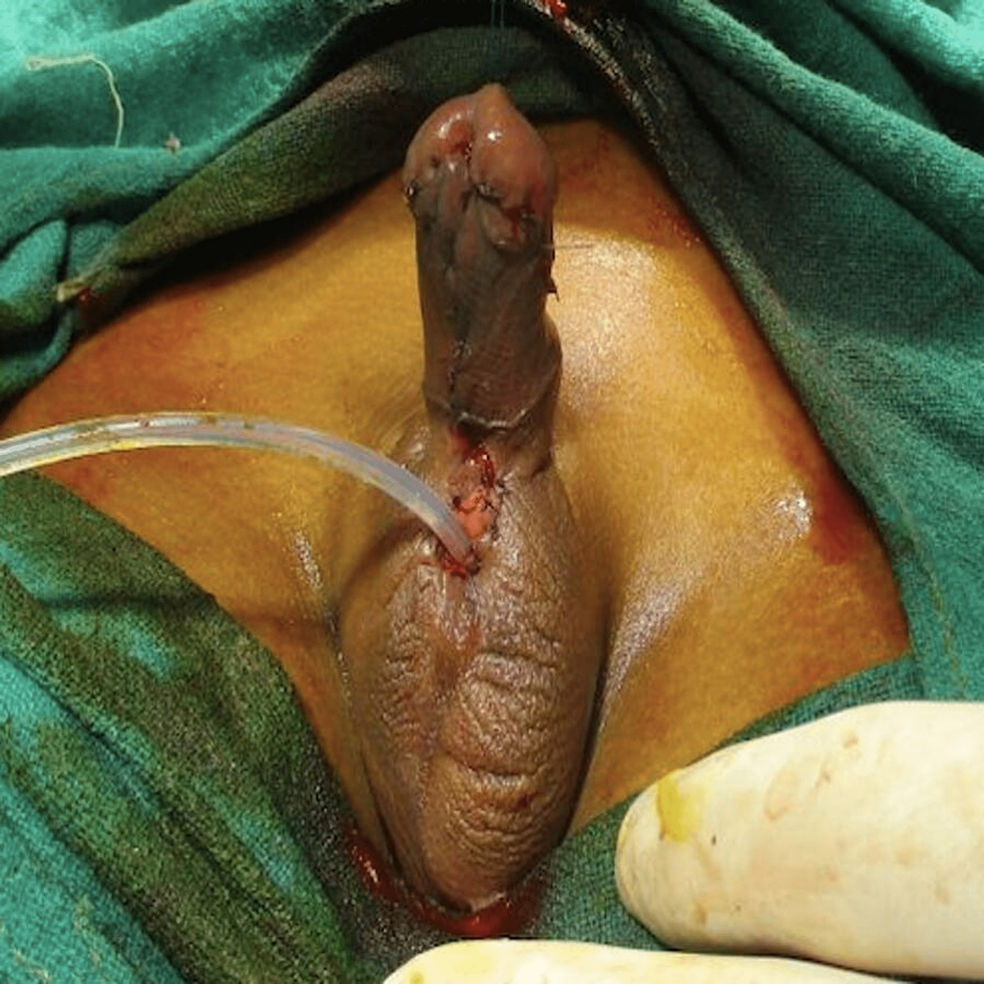
Intraoperative image of the child showing stage 1 hypospadias repair with release of chordee Captured by JV

To treat this child, a multidisciplinary approach was adopted wherein for the cleft lip, an oral surgery reference was taken where they advised for surgical repair. A pediatric surgeon's opinion was taken for hypospadias who suggested the surgical correction of hypospadias. After obtaining pediatric and cardiac fitness, the child was posted for stage 1 hypospadias repair with chordee release (Figure 4).

The step 1 surgical intervention was successful, and he was advised to follow up after six months for the second stage. Due to financial constraints, the patient could not be operated on for cleft lip and ventricular septal defect. The patient was discharged after day 6 of postoperative care and was advised for follow-up after four weeks and further management of the cleft lip and ventricular septal defect.

A written informed consent was obtained from the father for the publication of this case report and any accompanying photographs.

## Discussion

Telecanthus-hypospadias syndrome is an X-linked disorder where males are affected while females are carriers. The incidence of the syndrome among males is 1:50000-1:100000 [2]. Apart from X-linked inheritance, it is also expressed as an autosomal dominant inheritance. De Falco et al. studied mutations in the *MDR1* gene of 63 males and referred to them as familial or sporadic cases, found 11 novel mutations among 63 cases, and concluded that among all the *MDR1* gene mutations, the most frequent manifestation is hypospadias and hypertelorism, which were present in almost all cases of Opitz syndrome [4]. Even different affected members of the same family can express different phenotypic features. *MID1* mutations are responsible for the X-linked form of Opitz syndrome. It can involve both familial and sporadic cases. The mutation of the *MDR1* gene is scattered along the entire gene, which involves deletion, insertion, missense, and nonsense mutations, causing frameshifts involving the deletion of a single exon or entire *MID1* coding region [5].

Neurological manifestations such as Dandy-Walker malformation, hypoplasia, or agenesis of the corpus callosum and/or cerebellar vermis can also be present in telecanthus-hypospadias syndrome [2]. A multidisciplinary approach is needed for the management of a patient with telecanthus-hypospadias syndrome. Various procedures had been performed previously and advocated for the correction of hypertelorism such as the Young procedure, canthoplasty, box osteotomy, and subcranial osteotomy. For cleft lip and cleft palate, various procedures can be done such as lip repair, palate repair, alveolar bone graft, and pharyngoplasty. Other procedures can be done for the defect involved such as respiratory, cardiovascular, and imperforate anus. A multidisciplinary approach is of utmost importance to identify all the defects involved in the patient to manage properly with all the specialties involved [6]. Einfeld et al. reported the death of an eight-year-old male who had Opitz syndrome with feeding difficulty from birth to eight years of age [7]. He was assessed, and an endoscopy was performed for the examination of the pharynx, larynx, trachea, bronchi, and esophagus, which was normal with only possible abnormality of the left lower basal segment bronchus. He was having frequent admissions. He had an upper respiratory tract infection, followed by vomiting and massive aspiration. The autopsy showed hypoplasia of epiglottis with the narrowing of the trachea with no laryngotracheal-esophageal development defect. So all the upper respiratory and gastrointestinal symptoms of children with the syndrome should be monitored closely [7]. Some of the uncommon findings noted in this syndrome include nevi, dry itchy skin, pyloric stenosis, ileal atresia, multilobulated spleen, vertebral anomalies, megalocornea, and myopia [8].

In a study by Summit and Wilroy [9], where they evaluated eight families and 12 affected males, the spectrum of anomalies found in them included mental retardation, which affected eight males; cranial asymmetry, which affected as many as 11 out of 12 males; strabismus; cleft lip and palate; congenital heart disease, which affected six; abnormality of the urinary tract, which affected four; and cryptorchidism, which affected five. So, it is important to examine patients who have widely spaced ocular canthi and genital abnormalities so that genetic counseling can be provided. In 14 patients with telecanthus-hypospadias syndrome, Cassab et al. conducted an auditory examination and electrophysiological tests. They discovered that 12 of the patients had normal auditory thresholds and two had changed thresholds. When hearing loss occurs, the central auditory pathway is implicated in the brainstem, necessitating imaging studies [10]. As telecanthus-hypospadias syndrome has a wide range of signs and symptoms showing varied phenotypic variations in different patients ranging from mild to life-threatening conditions, therefore, it is imperative to have monitoring of symptoms in these patients.

## Conclusions

Telecanthus-hypospadias syndrome is a rare and life-threatening condition if the cardiovascular or respiratory involvement is significant. Therefore, whenever a patient with hypospadias has been examined, the evaluation of the cardiovascular, neurological, and respiratory systems should be done vigilantly to determine these abnormalities in the initial evaluation leading to its timely management. A multidisciplinary approach involving management at multiple steps on consulting all the concerned specialties has to be done.
